# Epstein-Barr Virus-Positive Plasmablastic Lymphoma Arising in the Anal Canal of an Immunocompetent Patient

**DOI:** 10.7759/cureus.89785

**Published:** 2025-08-11

**Authors:** Abdulrahman Al-Majmuei, Abdullah Al-Naqeeb, Farah Fakhri, Paul G Murray, Eman Aljuffairi

**Affiliations:** 1 Department of Pathology, School of Medicine, Royal College of Surgeons in Ireland - Bahrain, Muharraq, BHR; 2 Pathology and Laboratory Medicine, King Hamad University Hospital, Muharraq, BHR

**Keywords:** anal canal tumour, epstein-barr virus, immunocompetent patient, pathology case report, plasmablastic lymphoma

## Abstract

This case report describes a 57-year-old immunocompetent male diagnosed with Epstein-Barr virus (EBV)-positive plasmablastic lymphoma (PBL), a rare and aggressive subtype of diffuse large B-cell lymphoma (DLBCL), presenting in the anal canal. PBL is most commonly seen in immunocompromised patients, particularly those with a human immunodeficiency virus (HIV) infection; its presence in immunocompetent individuals is exceedingly rare. The patient presented with persistent anal pain, prompting a comprehensive diagnostic workup. Histopathological and immunohistochemical analysis confirmed PBL, with a high proliferation index and EBV-encoded RNA (EBER) positivity. The patient initially responded to dose-adjusted rituximab, etoposide, prednisone, vincristine, cyclophosphamide, and doxorubicin (DA-R-EPOCH) chemotherapy, with a reduction in mass size and metabolic activity. However, the disease relapsed, requiring further treatment with ifosfamide, carboplatin, and etoposide (ICE) chemotherapy, followed by consolidation with autologous stem cell transplantation (ASCT), after which the patient remained in remission for six months. This report not only discusses the clinical and pathological findings but also explores the interplay between EBV and the immune system in tumor development. The case underscores the need for heightened clinical suspicion and the future potential for immunotherapeutic and EBV-targeted therapies for patients with EBV-positive PBL.

## Introduction

Plasmablastic lymphoma (PBL) is a rare and aggressive subtype of diffuse large B-cell lymphoma (DLBCL), most commonly associated with human immunodeficiency virus (HIV) infection and other forms of immunosuppression. It is characterized by the expression of plasma cell markers such as cluster of differentiation 138 (CD138) and multiple myeloma oncogene 1 (MUM1), the absence of pan-B-cell markers like cluster of differentiation 20 (CD20) and paired box protein 5 (PAX5), a high proliferation index (Ki-67 >90%), and frequent association with Epstein-Barr virus (EBV) [1].

PBL accounts for approximately 2-3% of all HIV-associated lymphomas, but cases occurring in HIV-negative, immunocompetent individuals are exceedingly rare, with only a limited number reported in the literature [2]. The anal canal is an especially uncommon site of involvement, with only two such cases described in this population to date [3]. In this case, the patient was HIV-negative and had no history of immunosuppressive conditions such as diabetes mellitus, autoimmune disease, use of immunosuppressive medications, or any other known cause of immunosuppression, supporting an immunocompetent status. The detection of EBV in a tumor arising within an immunocompetent host underscores the virus’s ability to subvert host immune defenses, suggesting a more nuanced role for EBV-mediated immune modulation in the absence of overt immunosuppression. EBV may shape the tumor microenvironment through mechanisms that include altered cytokine signaling, immune checkpoint upregulation, and impaired antigen presentation [4,5].

In detailing the diagnostic and therapeutic course, this report also highlights the underlying immunovirological interactions that may inform future approaches to risk stratification and the development of targeted immunotherapies and antiviral treatments.

## Case presentation

In June 2023, a 57-year-old immunocompetent male from the Middle East presented with persistent anal pain lasting six months, accompanied by localized swelling. Physical examination revealed a firm, ulcerated anal mass and palpable left inguinal lymphadenopathy. Initial laboratory investigations were largely unremarkable, apart from a mildly decreased serum ferritin level, suggestive of iron deficiency, and a slightly low alanine aminotransferase (ALT), which was not considered clinically significant. A summary of the patient’s key laboratory results is shown in Table 1. A bone marrow biopsy was also performed to assess for systemic involvement and showed no evidence of lymphoma, confirming the absence of bone marrow infiltration at the time of diagnosis.

**Table 1:** Summary of key laboratory test results on initial presentation RBC: red blood cells; MCV: mean corpuscular volume; MCH: mean corpuscular hemoglobin; MCHC: mean corpuscular hemoglobin concentration; WBC: white blood cells; LDH: lactate dehydrogenase; ALP: alkaline phosphatase; ALT: alanine aminotransferase; AST: aspartate aminotransferase; GGT: gamma-glutamyltransferase

Test	Results	Unit	Reference range
RBC count	5.3	×10¹²/L	4.1-5.5
Hemoglobin	15.2	g/dL	13-18
Hematocrit	46.4	%	40-50
MCV	86.6	fL	80-100
MCH	28.4	pg	27-32
MCHC	32.8	g/dL	31-36
Ferritin	14.9	ng/mL	16-323
Transferrin saturation	27.8	%	15-33
WBC count	6.3	×10⁹/L	4-11
Platelet count	254.0	×10⁹/L	150-450
Albumin	46.8	g/L	38-50
LDH	178.0	U/L	100-190
Creatinine	80.5	umol/L	62-115
ALP	98.1	U/L	30-130
ALT	11.7	U/L	16-63
AST	18.0	U/L	15-40
GGT	21.6	U/L	15-85

**Figure 1:**
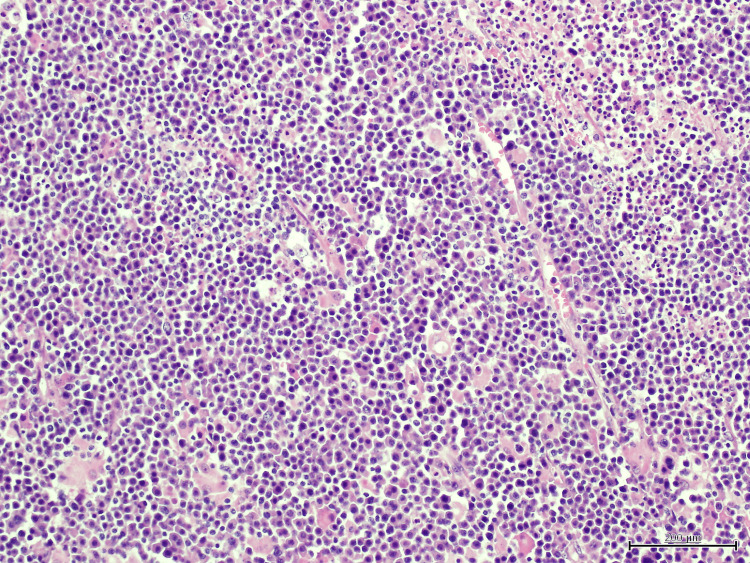
Hematoxylin and eosin (H&E) stain at 40x magnification showing sheets of large atypical lymphoid cells with prominent nucleoli, high mitotic activity, and areas of necrosis.

**Figure 2:**
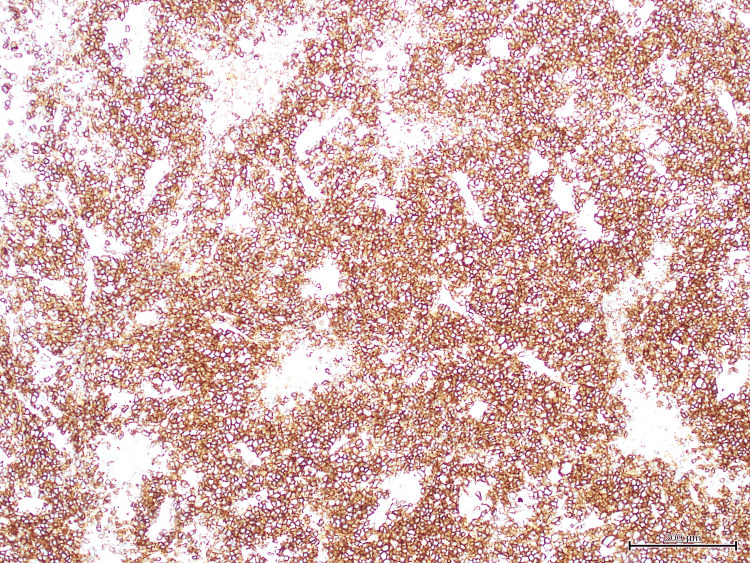
CD138 immunostaining showing strong diffuse membranous and cytoplasmic expression in the tumor cells, supporting plasmacytic differentiation.

**Figure 3:**
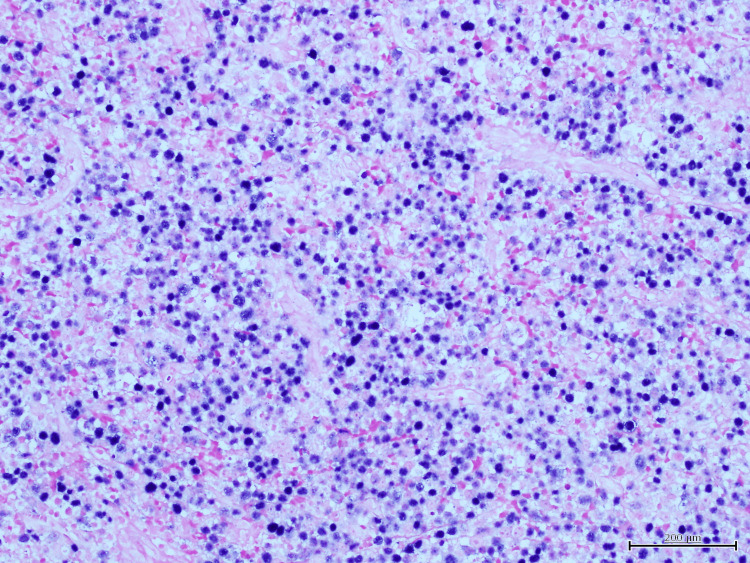
Epstein-Barr virus (EBER) in-situ hybridization demonstrating strong nuclear positivity in tumor cells, confirming Epstein-Barr virus association.

**Figure 4:**
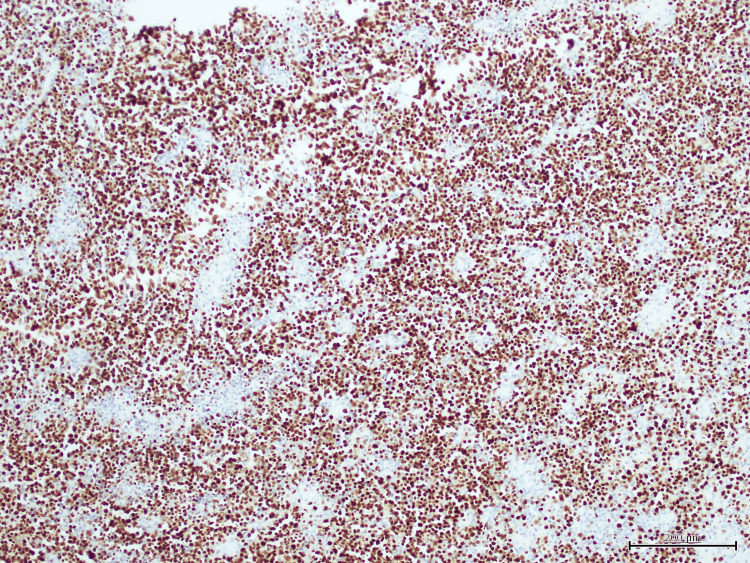
MUM1 immunohistochemistry showing diffuse nuclear positivity in tumor cells, further supporting plasma cell lineage.

**Figure 5:**
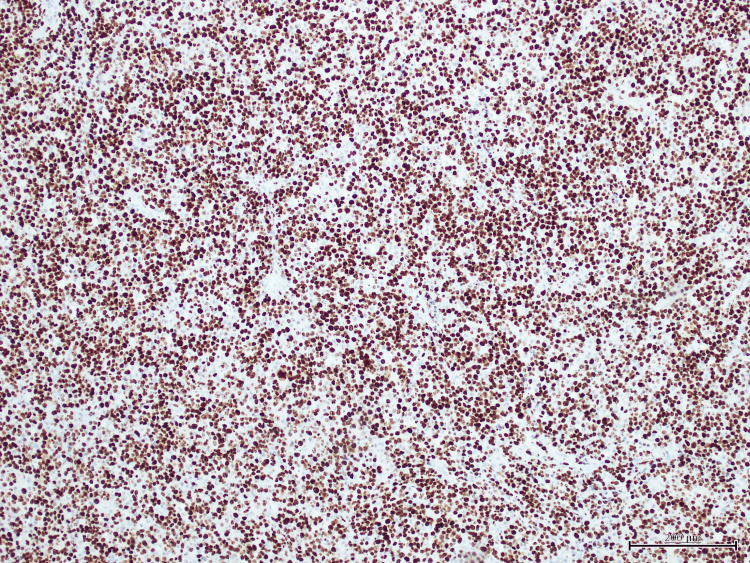
Ki-67 staining showing a high proliferation index of approximately 90%, indicating aggressive tumor biology.

Histopathological analysis of the anal mass showed a diffuse proliferation of large, atypical cells with plasmablastic features, including prominent nucleoli, high mitotic activity, and areas of necrosis (Figure 1). Immunohistochemistry revealed strong, diffuse membranous and cytoplasmic expression of CD138, consistent with plasmacytic differentiation (Figure 2). Epstein-Barr virus-encoded RNA (EBER) in-situ hybridization demonstrated strong nuclear positivity, confirming EBV association (Figure 3). Additional staining showed diffuse nuclear expression of MUM-1, further supporting plasma cell lineage (Figure 4). The Ki-67 proliferation index exceeded 90%, reflecting a highly proliferative and aggressive tumor phenotype, which is associated with poorer clinical outcomes in PBL [2] (Figure 5).

Staging positron emission tomography/computed tomography (PET/CT) revealed fluorodeoxyglucose (FDG)-avid mural thickening of the left lateral anal canal, with a maximum standardized uptake value (SUVmax) of 13.3. Multiple enlarged lymph nodes were also noted in the left obturator and iliac chains. No distant disease was identified, and the lymphoma was staged as Ann Arbor IIE. The patient was started on three cycles of dose-adjusted rituximab, etoposide, prednisone, vincristine, cyclophosphamide, and doxorubicin (DA-R-EPOCH) chemotherapy, to which there was an initial clinical response.

However, repeat PET/CT in December 2023 demonstrated disease progression in nodal sites, despite regression of the primary anal mass. New FDG-avid lymph nodes were observed in the left obturator, iliac, femoral, and aorto-caval regions. The most metabolically active node, located in the right common iliac area, measured 3.0 × 2.5 cm with an SUVmax of 29.5. Biopsy of one of the enlarged lymph nodes confirmed the recurrence of PBL. The patient subsequently underwent two additional cycles of DA-R-EPOCH, followed by ifosfamide, carboplatin, and etoposide (ICE) chemotherapy.

A follow-up PET/CT in April 2024 showed significant regression in both nodal size and FDG uptake, with the largest remaining node measuring 1.8 × 1.6 cm and an SUVmax of 2.9. The primary anal canal lesion remained in metabolic remission. In July 2024, the patient underwent autologous stem cell transplantation (ASCT) using carmustine, etoposide, cytarabine, and melphalan (BEAM) conditioning. As of January 2025, the patient remains in remission. A summary of the patient’s clinical timeline and treatment course is provided in Table 2.

**Table 2:** Summary of the patient’s clinical timeline and treatment course PET/CT: positron emission tomography/computed tomography; DA-R-EPOCH: dose-adjusted rituximab, etoposide, prednisone, vincristine, cyclophosphamide, and doxorubicin; ICE: ifosfamide, carboplatin, and etoposide; ASCT: autologous stem cell transplantation; BEAM: carmustine, etoposide, cytarabine, and melphalan

Date	Clinical event
June 2023	Initial presentation with anal pain and swelling. Anal mass and lymphadenopathy noted.
July 2023	Diagnosis of plasmablastic lymphoma confirmed by biopsy. PET/CT shows localized disease.
Aug-Oct 2023	Three cycles of DA-R-EPOCH chemotherapy administered. Initial clinical response observed.
December 2023	Disease progression on repeat PET/CT. Relapse confirmed by nodal biopsy.
Dec 2023-Feb 2024	Two more cycles of DA-R-EPOCH and ICE chemotherapy given.
April 2024	PET/CT shows nodal regression.
July 2024	ASCT performed with BEAM conditioning.
January 2025	The patient remains in remission.

## Discussion

This case of EBV-positive PBL in an immunocompetent host highlights the complex interplay between viral oncogenesis and host immune modulation. EBV contributes to lymphomagenesis through the expression of latent membrane protein 1 (LMP1), which constitutively activates several pro-oncogenic signaling cascades, including the nuclear factor kappa-light-chain-enhancer of activated B cells (NF-κB) pathway. LMP1 also inhibits apoptosis, while Epstein-Barr virus nuclear antigens (EBNAs) promote B-cell immortalization [6].

For EBV to persist in tumors arising in immunocompetent individuals, it must evade intact immune surveillance. This is accomplished through mechanisms such as downregulation of antigen presentation pathways, expression of viral microRNAs, and modulation of tumor-associated macrophages (TAMs) and regulatory T-cell (Treg) function [7]. While PBL cells typically lack surface immunoglobulins, they express plasma cell markers, indicating terminal B-cell differentiation. The high Ki-67 proliferation index observed in this case reflects an aggressive phenotype driven by uncontrolled cellular replication [8]. EBV further contributes by altering the tumor microenvironment in favor of immune tolerance, potentially leading to chemotherapy resistance and rapid disease progression [9].

Reported cases in immunocompetent individuals, including the present case, have exhibited aggressive clinical courses requiring intensive treatment [2,3]. This pattern indicates that preserved immune function does not necessarily translate into a more favorable prognosis in EBV-positive PBL and underscores the importance of maintaining a high index of suspicion even in patients without conventional risk factors.

Given the aggressive nature of PBL and its poor response to conventional treatment, there is growing interest in EBV-targeted therapies. These include the adoptive transfer of EBV-specific cytotoxic T lymphocytes (CTLs), immune checkpoint inhibitors such as programmed death-1 (PD-1)/programmed death-ligand 1 (PD-L1) blockade to enhance T-cell responses, and epigenetic modulators that upregulate viral antigen expression [10,11]. Such treatments may improve outcomes, particularly in relapsed or refractory EBV-positive lymphomas.

The decision to proceed with ASCT in this case was based on several high-risk features: aggressive relapse following initial therapy, a high proliferation index, and EBV positivity. Although the current data are limited, ASCT appears to improve survival in chemosensitive relapsed PBL [12]. The optimal timing of ASCT, whether during first remission or after relapse, remains an open question in immunocompetent patients with PBL.

This case emphasizes the importance of assessing EBV status in atypical lymphomas, even in patients without known immunosuppression. It also underscores the role of translational immunology in guiding future treatment strategies, particularly the integration of virus-targeted and immune-modulating therapies into standard lymphoma care. Recent molecular profiling indicates that EBV-positive PBL harbors alterations in key signaling pathways, including Janus kinase/signal transducer and activator of transcription (JAK-STAT), phosphoinositide 3-kinase/protein kinase B (PI3K-AKT), and mitogen-activated protein kinase/extracellular signal-regulated kinase (MAPK-ERK), offering further insight into viral oncogenesis and identifying promising therapeutic targets [6]. In addition, EBV-associated viral microRNAs may repress pro-apoptotic genes and impair antigen-processing machinery, contributing to an immunologically “cold” microenvironment even in immunocompetent individuals [6,7]. These findings suggest that EBV plays a role not only in tumor initiation but also in immune evasion and resistance to treatment.

## Conclusions

This case highlights the diagnostic and therapeutic challenges of EBV-positive PBL, particularly when it arises in atypical sites like the anal canal and in immunocompetent individuals without traditional risk factors. It underscores the importance of maintaining clinical suspicion, performing early biopsy, and testing for EBV in persistent anorectal lesions, even in patients without immunodeficiency. The findings suggest that preserved immune status does not guarantee a favorable prognosis, and that early recognition, followed by chemotherapy and consideration of ASCT, remains critical for timely and effective intervention.
